# Clinical considerations in transitioning patients with epilepsy from clonazepam to clobazam: a case series

**DOI:** 10.1186/1752-1947-8-429

**Published:** 2014-12-16

**Authors:** Raman Sankar, Steve Chung, Michael Scott Perry, Ruben Kuzniecky, Saurabh Sinha

**Affiliations:** 11 Division of Pediatric Neurology, David Geffen School of Medicine at UCLA, University of California–Los Angeles, Room 22-474 MDCC, Los Angeles, CA 90095-1752 USA; 12 Barrow Neurological Institute, 500 W. Thomas Road, Phoenix, AZ 85013 USA; 13 Jane and John Justin Neuroscience Center, Cook Children’s Medical Center, 4th Floor, 1500 Cooper Street, Fort Worth, TX 76104 USA; 14 Department of Neurology, NYU Epilepsy Center, New York University School of Medicine, 223 East 34 Street, New York, NY 10016 USA; 15 Neurology Residency Program, Epilepsy Monitoring Unit, Duke University Medical Center, DUMC 102350, Durham, NC 27710 USA

**Keywords:** Clobazam, Clonazepam, Switching, Transition

## Abstract

**Introduction:**

In treating refractory epilepsy, many clinicians are interested in methods used to transition patients receiving clonazepam to clobazam to maintain or increase seizure control, improve tolerability of patients’ overall drug therapy regimens, and to enhance quality of life for patients and their families. However, no published guidelines assist clinicians in successfully accomplishing this change safely.

**Case presentations:**

The following three case reports provide insight into the transition from clonazepam to clobazam. First, an 8-year-old Caucasian boy with cryptogenic Lennox–Gastaut syndrome beginning at 3.5 years of age, who was experiencing multiple daily generalized tonic–clonic, absence, myoclonic, and tonic seizures at presentation. Second, a 25-year-old, left-handed, White Hispanic man with moderate mental retardation and medically refractory seizures that he began experiencing at 1 year of age, secondary to tuberous sclerosis. When first presented to an epilepsy center, he had been receiving levetiracetam, valproate, and clonazepam, but reported having ongoing and frequent seizures. Third, a 69-year-old Korean woman who had been healthy until she had a stroke in 2009 with subsequent right hemiparesis; as a result, she became less physically and socially active, and had her first convulsive seizure approximately 4 months after the stroke.

**Conclusions:**

From these cases, we observe that a rough estimate of final clobazam dosage for each mg of clonazepam under substitution is likely to be at least 10-fold, probably closer to 15-fold for many patients, and as high as 20-fold for a few. Consideration and discussion of the pharmacokinetic, pharmacologic, and clinical properties of 1,4- and 1,5-benzodiazepine action provide a rationale on why and how these transitions were successful.

## Introduction

Benzodiazepines have been used to treat seizures since 1965, when a group from Marseilles, led by Henri Gastaut, reported on the successful use of diazepam to treat non-convulsive (mainly absence) seizures [1] and its utility in the treatment of status epilepticus [2]. Prior to that, benzodiazepines such as diazepam and chlordiazepoxide had been employed primarily as anxiolytics and hypnotics. Despite the observations by Gastaut, the use of diazepam for absence epilepsy did not gain traction because the 1960s marked the emergence of succinimides such as ethosuximide, which seemed to be less sedating and better tolerated than diazepam. Subsequently, Henri Gastaut published that clonazepam was even more effective than diazepam in the treatment of status epilepticus [3].

The initial approval of clonazepam by the US Food and Drug Administration (FDA), as reviewed by Thomas Brown [4], suggested that it be used for the following types of seizures: absence (typical petit mal), infantile spasms (infantile myoclonic, massive spasms, salaam), atypical absence (atypical petit mal), akinetic (astatic, atonic, drop attack), “minor motor” attacks, and “Lennox–Gastaut syndrome,” (LGS) but not for the treatment of grand mal, partial seizures with complex symptoms (for example, psychomotor, temporal lobe), or focal seizures. The more recent version of the prescribing information is more restrictive and states that, “It is useful alone or as an adjunct in the treatment of the LGS (petit mal variant), akinetic and myoclonic seizures. In patients with absence seizures (petit mal) who have failed to respond to succinimides, clonazepam (Klonopin™) may be useful [5].” Of interest, the initial experience described in the European literature was based on the use of intravenous clonazepam, which is not available in the USA.

Until the approval of clobazam (Onfi™) in the USA in October 2011 for adjunctive treatment of seizures associated with LGS [6], benzodiazepines had been used mainly as rescue medications for status epilepticus or acute repetitive seizures. In addition to concerns about excessive sedation, long-term benzodiazepine use raises questions about maintenance of efficacy because of potential development of tolerance. The indications section of the FDA prescribing information for clonazepam indicates, “In some studies, up to 30% of patients have shown a loss of anticonvulsant activity, often within 3 months of administration. In some cases, dosage adjustment may re-establish efficacy [5].”

In addition, of the various classes of anticonvulsant medications, benzodiazepines have been especially implicated in producing cognitive and behavioral adverse effects [7, 8]. However, clobazam, a 1,5-benzodiazepine, seems to differ in its spectrum of pharmacologic activities from traditional 1,4-benzodiazepines [9, 10]. Indeed, a recent study provided sustained efficacy results for clobazam in patients with LGS over 3 years with stable dosing [11].

Clinicians are considerably interested in switching some patients receiving clonazepam to clobazam to maintain or improve seizure control, while also improving tolerability and quality of life for the patient. However, no published guidelines assist clinicians in successfully accomplishing this change safely. In this report, we provide three case reports on this therapy transition, and summarize in the following discussion the known pharmacokinetic, pharmacologic, and clinical aspects of benzodiazepine action to arrive at a successful therapeutic solution to this problem.

## Case presentations

### Case 1 (8-year-old Caucasian boy with cryptogenic LGS)

Case 1 is an 8-year-old Caucasian boy with cryptogenic LGS beginning at 3.5 years of age. An electroencephalograph (EEG) showed diffuse slowing, with a generalized slow spike-and-wave pattern typical of LGS. His magnetic resonance imaging (MRI) was normal. At the time of presentation, he experienced multiple daily generalized tonic–clonic (GTC), absence, myoclonic, and tonic seizures. He had failed several prior antiepileptic drugs including levetiracetam (maximum dosage unknown), lamotrigine (7mg/kg/day), the ketogenic diet, and vagal nerve stimulation (VNS, output current 2.5mA, on time: 21 seconds, off time: 0.8 minutes). A physical examination revealed a thin boy, unable to walk or speak with many healing abrasions from frequent seizure-induced falls. At the time clobazam was added to his treatment regimen, he was failing zonisamide (10mg/kg/day), valproate (42mg/kg/day, concentration 103μg/mL), rufinamide (50mg/kg/day), and clonazepam 1.5mg twice daily.

Clobazam was initiated at 5mg daily (0.25mg/kg/day) and increased weekly by 5mg until he reached 10mg twice daily (1mg/kg/day) over 4 weeks. At this dosing, he became free of tonic and myoclonic seizures, with more than a 90% reduction of GTC and absence seizures. However, his parents reported significant somnolence, with only 6 hours of wake time each day. Clonazepam weaning was initiated by decreasing dosage by 0.5mg each week, until clonazepam was entirely discontinued by the end of 6 weeks. As clonazepam was weaned, somnolence resolved, and he continued the same good seizure control.

Rufinamide was subsequently discontinued without incident. Clobazam was titrated to 20mg twice daily (2mg/kg/day) 2 years after initiation and secondary to recurrent GTC seizures, which occurred in the setting of VNS dysfunction. Now, 3 years after the initiation of clobazam, he experiences rare myoclonic seizures and is able to walk independently, attend school, and communicate with his family.

### Case 2 (25-year-old, left-handed, White Hispanic man with moderate mental retardation and medically refractory seizures)

Case 2 is a 25-year-old, left-handed, White Hispanic man with moderate mental retardation and medically refractory seizures that he began experiencing at age 1 year, secondary to tuberous sclerosis. His seizures commence typically with sudden body stiffening for 30 seconds with ictal cry, followed by tonic–clonic seizure, on occasion. Seizure frequency had increased over time, and he often experienced seizures three to four times per day. He had been treated previously with carbamazepine, phenytoin, phenobarbital, lamotrigine, and topiramate. Approximately 5 years ago, he underwent vagus nerve stimulator implantation, which improved his seizures only slightly.

When he first presented to an epilepsy center, he had been receiving levetiracetam, valproate, and clonazepam, but reported having ongoing and frequent seizures. His neurologic examination showed moderate cognitive difficulty, slurred speech, and mild right-arm weakness. Further, his right hand was smaller than his left. He was able to ambulate on his own, but swayed left and right with wide-base stance. His previous EEG showed diffuse background slowing, which was focally more in the left hemisphere. Interictal epileptiform discharges were detected mainly from the left frontotemporal region, along with occasional sharp discharges from the right hemisphere. His brain MRI showed subependymal nodules bilaterally and transmantle dysplasia over the left parietal lobe. Initial scalp ictal EEG indicated diffuse bilateral seizure onset during his initial body stiffening. However, better ictal progression was observed over the left hemisphere just prior to the progression of tonic–clonic phase. A subsequent intracranial grid electrode guided study revealed diffuse seizure onset, but maximally over the left frontal and parietal lobes near the transmantle dysplasia.

He underwent surgical resection of the dysplasia, but his seizures did not improve significantly. Because of his frequent seizures, clobazam was started and gradually replaced clonazepam, as indicated in Table 1.

**Table 1:** **Dosing transition from clonazepam to clobazam in a 25-year-old, left-handed White Hispanic man with moderate mental retardation and medically refractory seizures**

Week 0	Clonazepam 1mg BID
Week 1	Clonazepam 0.5mg BID; Clobazam 5mg qHS
Week 2	Clonazepam 0.5mg qHS; Clobazam 5mg BID
Week 3	Clonazepam discontinued; Clobazam 5mg qAM and 10mg qPM

BID, twice daily; qAM, every morning; qHS, at bedtime; qPM, every night.

His family reported that his seizure improvement was notable at Week 2, and his seizures became milder and less frequent (>80% reduction), without significant adverse effects from clobazam. During the next 6 months, his clobazam dosage was gradually increased to 15mg twice daily. His seizure frequency further improved to one to two per week. Moreover, 1 month after his clobazam dosage increase, his levetiracetam dosage was decreased by 50%. He and his family are quite happy with the improvement, and had reported that he has become more active.

### Case 3 (69-year-old Korean woman with convulsive seizures)

Case 3 is a 69-year-old Korean woman who had been quite healthy until she had a stroke in 2009, with subsequent right hemiparesis. Following her stroke, she became less active physically and socially, and approximately 4 months after the stroke, she had her first convulsive seizure. Since her first seizure, she has also noted balance difficulty and worsening of right-leg weakness, requiring use of a walker and a wheelchair. She was placed on phenytoin, and later on oxcarbazepine. Because of imbalance, oxcarbazepine was changed to levetiracetam. On her subsequent visit, her friend reported significant mood changes and anger outbursts, and she was changed from levetiracetam to lamotrigine gradually. She did not have recurrent seizures with lamotrigine.

However, because of worsening of dizziness at 100mg daily, lamotrigine was changed to valproate. Her balance issues improved, but she developed hand tremors and recurrent seizures, which often started with olfactory auras, anxiety, and confusion, typically lasting 1 to 2 minutes. On her following clinic visit, she reported another convulsive seizure, which resulted in falling to the ground. In addition, she continued to experience two to three seizures per week. Her MRI scan showed large encephalomalacia over the left frontoparietal region, and an EEG showed left-sided interictal epileptiform discharges, superimposed on background slowing over the left hemisphere. Primarily to address her anxiety, which at times could be associated with her seizures, she was started on clonazepam (1mg/day). Although her anxiety and seizures had improved, she was not able to tolerate clonazepam, often experiencing significant daytime drowsiness. Next, her clonazepam therapy was gradually replaced by clobazam over 2 weeks as described in Table 2.

**Table 2:** **Dosing transition from clonazepam to clobazam in a 69-year-old Korean woman who had experienced a stroke in 2009 and subsequent right hemiparesis leading to convulsive seizures**

Week 0	Clonazepam 1mg qHS
Week 1	Clonazepam 0.5mg qHS;, Clobazam 5mg qHS
Week 3	Clonazepam discontinued; Clobazam 10mg qHS

qHS, at bedtime.

With clobazam 10mg/day, she felt much better cognitively and reported seizure freedom for 3 months initially. Subsequently, she reported approximately one aura every 2 months, and she decided to continue her clobazam dosage without further increase. She did not have significant adverse effects, but felt transient somnolence for the first 2 weeks of her medication transition. She has reported doing much better with balance and seizure control.

## Discussion

The three cases represent distinct patterns of a clonazepam to clobazam transition. In the first case, the addition and titration of the clobazam dosage preceded the weaning of clonazepam. In the second and third cases, clonazepam was safely weaned as clobazam was added and titrated simultaneously. Each case highlights both improved seizure control as well as decreased adverse effects following the change from clonazepam to clobazam.

A study published in 1982 compared the preclinical efficacy and the protective index (PI = toxic dose in 50% of the population/effective dose in 50% of the population) of clobazam to those of diazepam, valproate, and phenobarbital and concluded that clobazam performed superiorly in rodent models of induced seizures [12]. Similar advantages for clobazam over 10 different 1,4-benzodiazepines were documented in a study of both anxiolytic performance and antiseizure effect, as measured by electroshock convulsions in rodents compared with sedation and myorelaxant properties [13]. However, this advantage in PI was not discernible in the kindling model [14].

**Figure 1:**
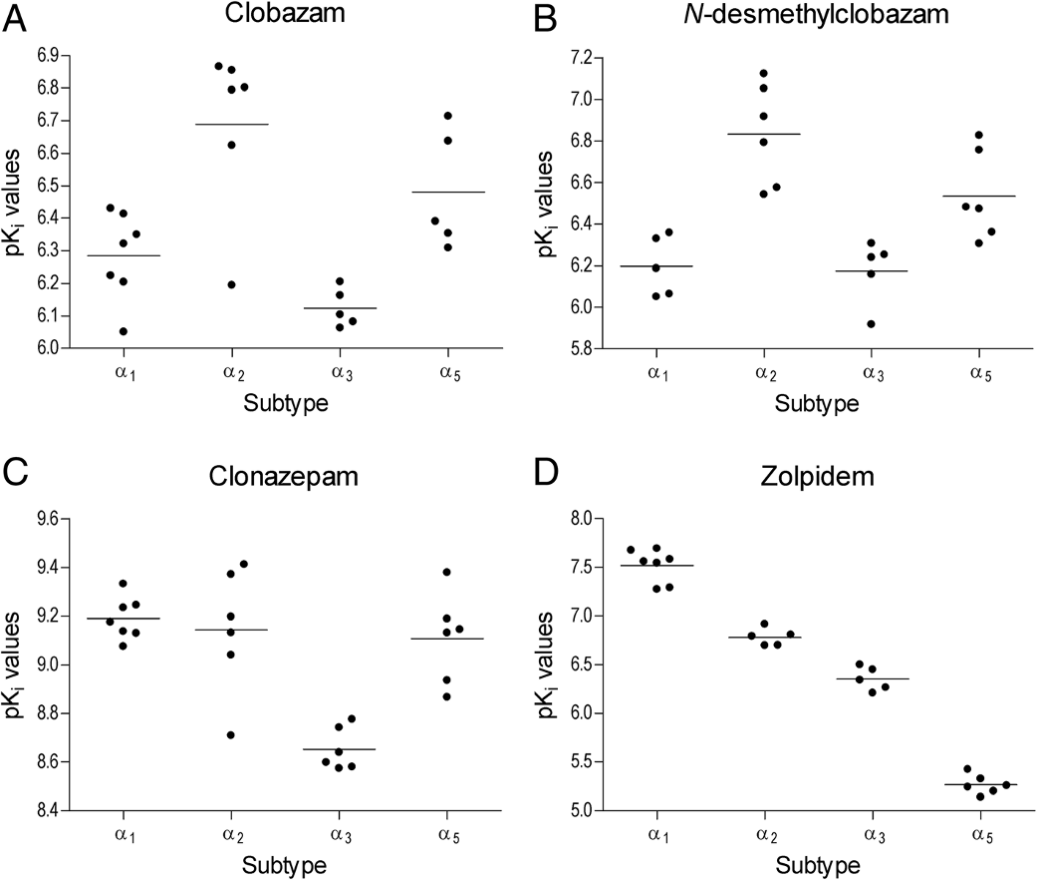
**Distribution of individually determined pK**_**i**_ **values for (A) clobazam, (B) N-desmethylclobazam, (C) clonazepam, and (D) zolpidem across GABAA-receptor subtypes.** From Jensen *et al*. [10]. K_i_, inhibition constant.

The advantages of clobazam over diazepam in human adaptive performance and reaction time have been studied [15, 16]. Nicholson suggested, based on these studies, that diazepam and its hydroxylated metabolites (temazepam, oxazepam, and nordiazepam) were suitable for use as hypnotics, while clobazam could be a better daytime anxiolytic [16]. In fact, the binding of clobazam and its metabolite, *N*-desmethylclobazam, to γ-aminobutyric acid (GABA) receptors in *in vitro* expression systems demonstrates that they have significantly greater binding affinities for α_2_- versus α_1_-subunit-containing GABA receptor complexes, a difference not observed for clonazepam, for which no distinction between α_2_ and α_1_ receptors was observed (Figure 1) [10]. Many lines of evidence suggest that GABA receptors containing α_1_ subunits mediate sedation (as is known for zolpidem), while substantial anticonvulsant activity can be achieved by activation of GABA receptors containing α_2_ subunits [9].

For development of tolerance, there appears to be differences among species and models compared with humans with epilepsy. Thus, in amygdala-kindled rats, tolerance to clobazam developed more rapidly than tolerance to clonazepam [17]. In acute seizures induced by pentylenetetrazole or maximal electroshock, rats chronically treated with diazepam, clonazepam, or clobazam for 28 days and tested over the ensuing 2 weeks showed similar extents of tolerance [18].

In a prospective Canadian study of 115 children with varying types of epilepsy treated with clobazam, the 79 responders developed at least some degree of tolerance over a 24-month period [19]. The same study also reported that 25 of 37 (68%) children who had failed to respond to 1,4-benzodiazepines (that is, nitrazepam or clonazepam) had responded to the 1,5-benzodiazepine clobazam. In addition, the use of clobazam appeared to be associated with less neurotoxicity than was observed for the 1,4-benzodiazepines clonazepam and nitrazepam. Another Canadian study, involving adult patients with different types of epilepsy, observed that those who had achieved a sustained response to clobazam differed from those who had developed tolerance in duration of epilepsy and the clobazam concentrations that had been maintained [20]. Sustained responders had experienced a shorter duration of epilepsy (mean 16.5 versus 24.5 years, p=0.015) and had attained greater clobazam concentrations (0.50 versus 0.22μM, p=0.017).

A human study followed patients who participated in Phase II and III LGS studies into an extended open-label phase. Of 74 patients who had achieved at least a 50% reduction in drop seizures from baseline to Month 3, 64 (86%) maintained this degree of response with stable dosages over time for up to 3 years [11]. In a subset of patients in that study for whom data were available for up to 5 years, the mean modal clobazam dosage was 0.97mg/kg/day compared with 0.90mg/kg/day in the first year. The retention rate for US patients at 2 years was nearly 80% [11]. Similar results were found in a single-center study of clobazam with children from Seoul, Korea, with LGS, which reported retention rates of 80.2% at 2 years and 76.6% at 3 years [21].

The switch from clonazepam to clobazam should be considered in light of relevant pharmacokinetic and pharmacodynamic factors of these compounds. One of the important determinants of brain uptake is relative lipophilicity, often estimated by the octanol-to-water partition ratios. Via this *in vitro* measure, clobazam is much less lipophilic than diazepam (ratio of 9 for clobazam versus 309 for diazepam). Similar data for clonazepam are not readily available [22, 23]. However, the retention times for diazepam, clonazepam, and clobazam in a reverse-phase, high-performance liquid chromatography (HPLC) system using a neutral mobile phase are 31.4 minutes, 14.9 minutes, and 13.1 minutes, suggesting that clonazepam and clobazam are fairly similar in this measure of lipophilicity. The retention time for *N*-desmethylclobazam is 12, again not far from the times for clonazepam or clobazam. Of the *in vitro* estimates, available data [22, 23] suggest that HPLC retention times correlated better with *in vivo* unbound volumes of distribution for a series of benzodiazepines. Arendt and colleagues [22] showed that despite the difference in lipophilicity, the cerebrospinal fluid-to-plasma ratio for clobazam was greater than that for diazepam, a result they attributed to the fact that clobazam is less protein-bound than diazepam (83% for clobazam versus 99% for diazepam), since it is the unbound fraction that transits the blood–brain barrier. Unfortunately, data were not provided for clonazepam in this report. The protein binding of clonazepam is reported to be 85% in a different article [24], and these values are also in agreement with those reported by Riss *et al*. [25]. Thus, based on a combination of lipophilicity and protein binding, the central nervous system penetration of clonazepam and clobazam should be comparable.

The binding affinities (K_i_ in nM) for clonazepam, clobazam, and *N*-desmethylclobazam to rat brain homogenates have been determined to be 0.26, 151, and 153 [10], respectively, corresponding to the greater potency of clonazepam over clobazam on a mg basis as used in patients. In the same report, Jensen *et al*. also showed that the pK_i_ values for clobazam and *N*- desmethylclobazam for α_2_-subunit-containing receptors exceeded that for those containing α_1_ subunits, whereas these values were similar for clonazepam. Zolpidem, however, exhibited the greatest pK_i_ values for receptors containing α_1_ subunits. These data would explain, at least in part, why clobazam may be much less sedating than clonazepam, as observed by Nicholson [16] nearly 35 years ago.

For drug disposition, clobazam (estimated half-life of 36 hours) undergoes hepatic metabolism to *N*-desmethylclobazam (estimated half-life of 80 hours). Because of the lower clearance, the latter achieves an approximately 10- to 15-fold steady concentration by approximately 4 weeks after initiation of therapy [26]. Both clobazam and *N*-desmethylclobazam show a bimodal response to chloride conductance when applied to cultured cerebral neurons in the presence of 10-μM GABA, peaking around 3μM clobazam or *N*-desmethylclobazam, and declining substantially at 10μM concentrations of either allosteric agonist [27]. Thus, under clinical conditions, *N*- desmethylclobazam can appear less potent. The important finding, however, is that the concentration of *N*-desmethylclobazam stabilizes 4 weeks after initiation of therapy. A study of drug–drug interactions found no clinically meaningful drug–drug interactions between clobazam and drugs metabolized by CYP3A4, CYP2C19, CYP1A2, or CYP2C9 [28]. The authors concluded that clobazam may be administered safely as adjunctive therapy in patients with LGS, without meaningful changes in clobazam pharmacokinetics that would require dosage adjustments.

## Conclusions

The literature reviewed thus supports the transition strategies highlighted in the cases described. A rough estimate of the final dosage of clobazam for each mg of clonazepam under substitution is likely to be at least 10-fold, probably closer to 15-fold for many patients, and as high as 20- fold for a few. While there is prudent caution in delaying the weaning of clonazepam until significant clobazam is already onboard from the point of view of breakthrough seizures, patients – as well as their families and caregivers – should be counseled to expect increased sedation transiently with that approach. Given the common clinical experience with the severity of withdrawal seizures associated with removal of clonazepam, it would be wise not to begin aggressive removal of clonazepam from the patients’ regimens as soon as clobazam is initiated. These guidelines are general, because if the patient is also receiving other medications acting via GABA mechanisms, the removal of clonazepam is likely to be tolerated slightly better and breakthrough seizures are less likely or may be milder should such seizures intervene. A transition from clonazepam to clobazam, in consideration of patients’ individual seizure patterns and vulnerabilities, with careful application of the principles described in this article, and with close clinical observation, can be reasonably expected to improve seizure control and enhance quality of life for patients and their families.

## Consent

Written informed consent was obtained from both of the adult patients and the parents of the child for publication in this case series. Copies of the written consents are available for review by the Editor-in-Chief of this journal.

## Abbreviations

CYP1A2: Cytochrome P450 isozyme 1A2
CYP2C9: Cytochrome P450 isozyme 2C9
CYP2C19: Cytochrome P450 isozyme 2C19
CYP3A4: Cytochrome P450 isozyme 3A4
EEG: Electroencephalograph
FDA: Food and drug administration
GABA: γ-aminobutyric acid
GTC: Generalized tonic–clonic
HPLC: High-performance liquid chromatography
K_i_: Inhibition constant
LGS: Lennox–Gastaut syndrome
MRI: Magnetic resonance imaging
PI: Protective index
VNS: Vagal nerve stimulation.
